# Supplementary figures and images for: Identifying conserved molecular targets required for cell migration of glioblastoma cancer stem cells
Source: Cell Death Dis. 2020 Feb 26;11(2):152. doi: 10.1038/s41419-020-2342-2 (PMC7044427; doi:10.1038/s41419-020-2342-2)

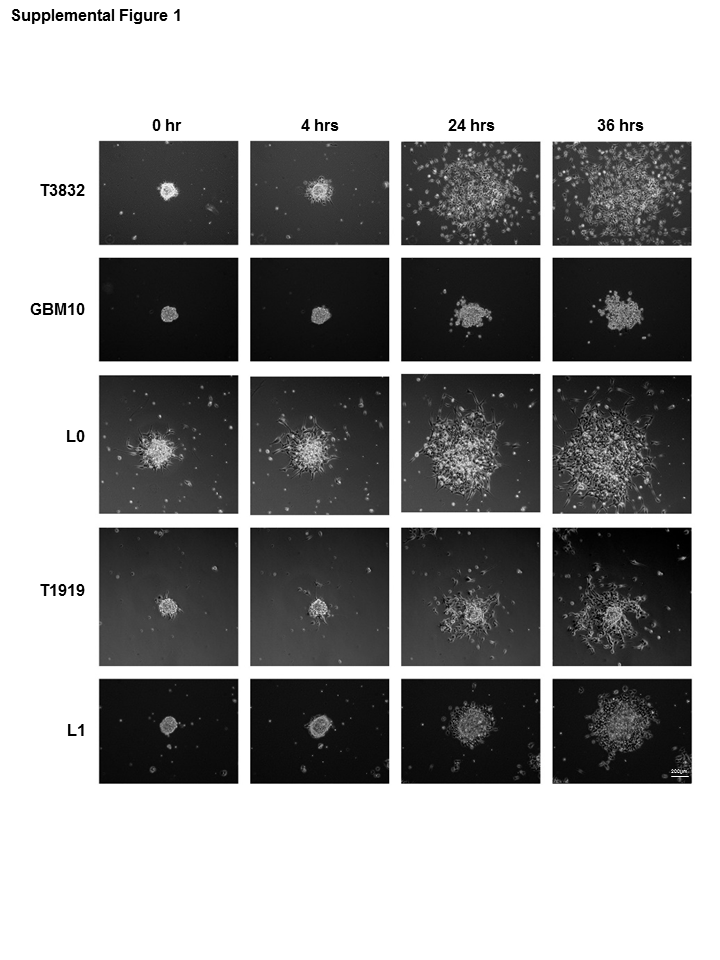

Supplement: Supplementary file 1 — Supplemental Figure 1 [file 41419_2020_2342_MOESM1_ESM.tif]

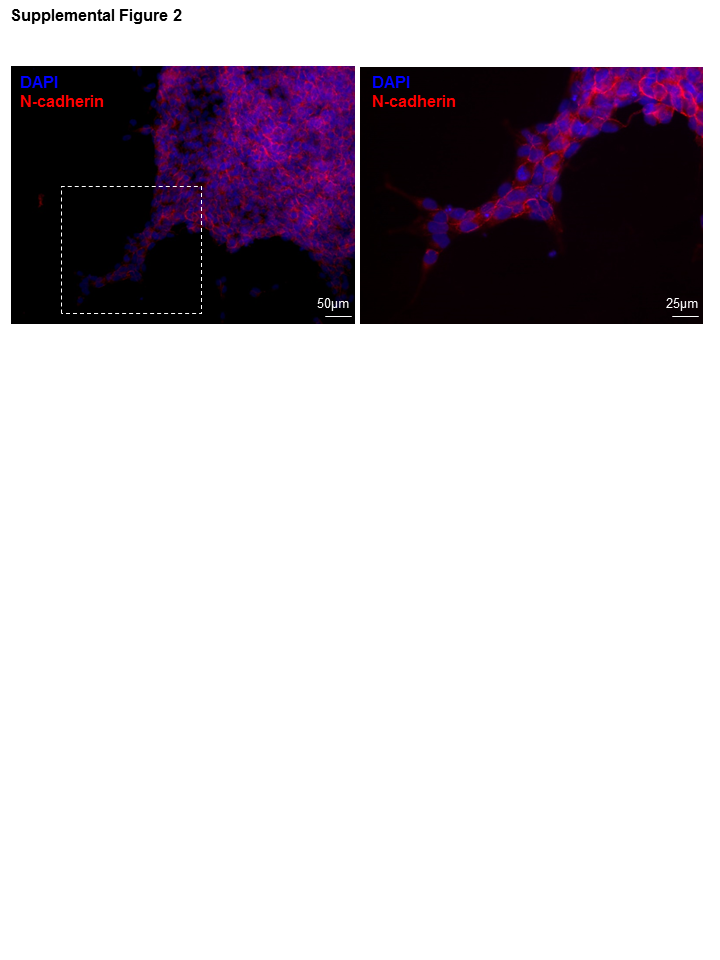

Supplement: Supplementary file 2 — Supplemental Figure 2 [file 41419_2020_2342_MOESM2_ESM.tif]

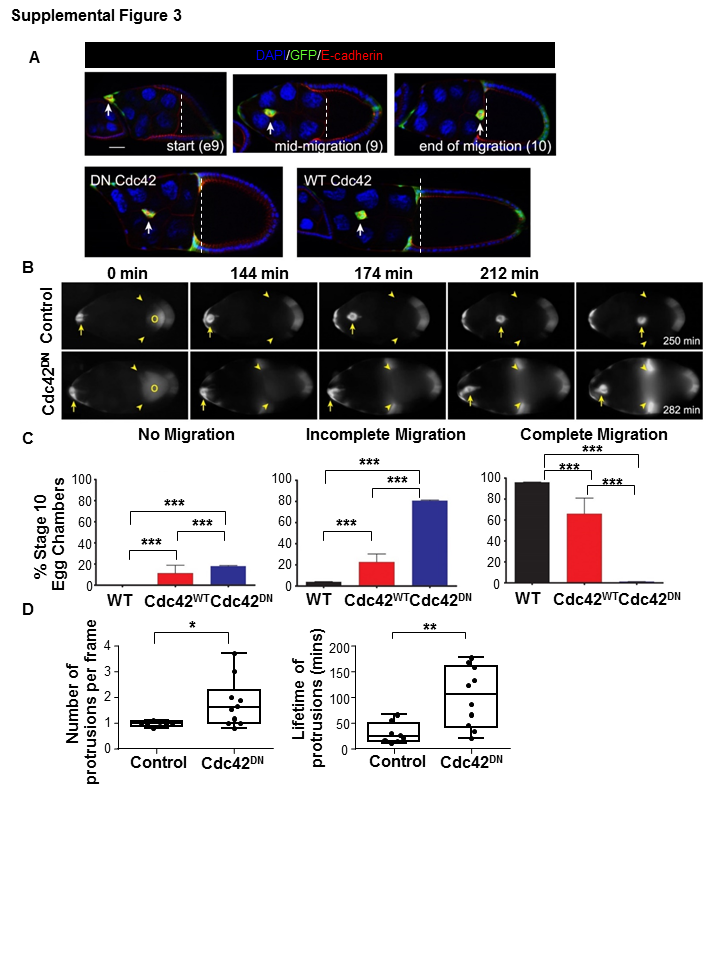

Supplement: Supplementary file 3 — Supplemental Figure 3 [file 41419_2020_2342_MOESM3_ESM.tif]

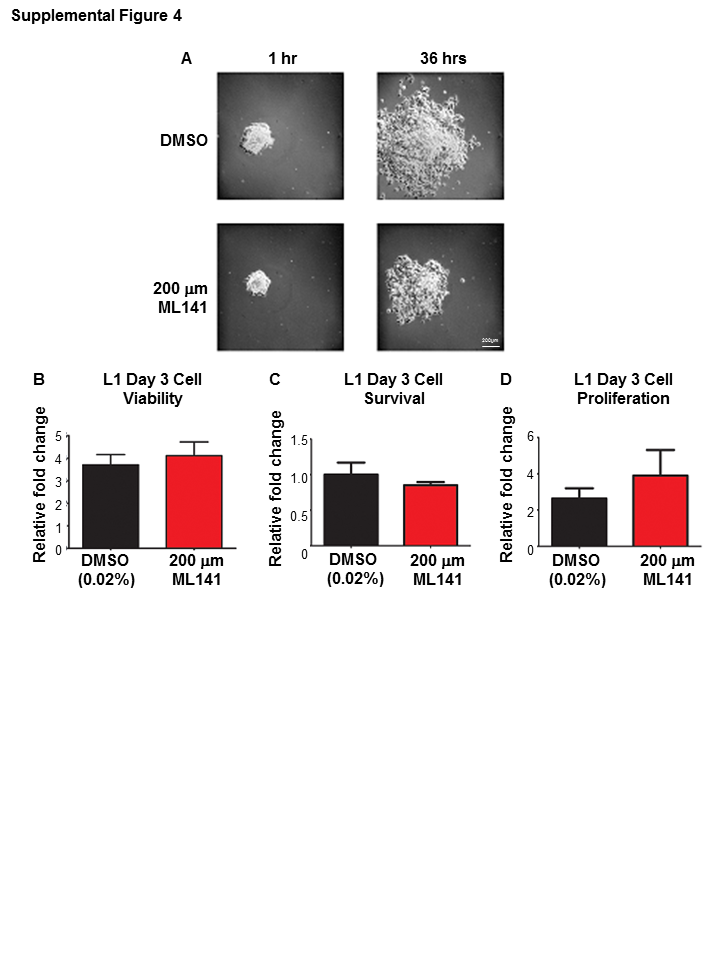

Supplement: Supplementary file 4 — Supplemental Figure 4 [file 41419_2020_2342_MOESM4_ESM.tif]
