# Supplementary material for: Identifying conserved molecular targets required for cell migration of glioblastoma cancer stem cells
Source: Cell Death Dis. 2020 Feb 26;11(2):152. doi: 10.1038/s41419-020-2342-2 (PMC7044427; doi:10.1038/s41419-020-2342-2)
Supplement: Supplementary file 6 — Supplemental Figures 1–4 [file 41419_2020_2342_MOESM6_ESM.docx]

**Supplemental Figure 1. GBM CSC lines exhibit a variety of migration modes.** Time-lapse microscopy of cells from patient-derived GBM CSC models (T3832, GBM10, L0, T1919, L1) exiting from a sphere over 36 hours (hrs).

**Supplemental Figure 2. N-cadherin localization in T387 tumorspheres.** Confocal micrographs of CSCs (T387) migrating as a collective out of a tumorsphere stained with an antibody against N-cadherin (red, left panel). Closer view of peripheral migrating finger-like projection (in box) is shown (right panel). Nuclei stained with 4′,6-diamidino-2-phenylindole (DAPI, blue). Scale bars represent 50μm and 25μm as indicated on individual micrographs.

**Supplemental Figure 3. Cdc42 levels correlate with border cell migration.** Cdc42 requirement during border cell collective migration (**A-D**). Control border cells (arrow) are shown at different stages of migration (top row), from early stage 9 (e9) at the start of migration up to stage 10 when they reach the oocyte. Expression of wildtype (WT) or dominant-negative (DN) Cdc42 in border cells (bottom row) using *slbo*-GAL4 both result in migration defects, as shown by the failure to reach the oocyte by stage 10. *slbo*-GAL4 was used to drive UAS-mCD8:GFP (green) expression in border cells; E-cadherin (red) labels all cell membranes; nuclei were visualized by DAPI (blue). Scale bar represents 20 μm (**A**). Stills from time-lapse movies of control border cells (top row) or border cells expressing DN Cdc42 (Cdc42^DN^, bottom row) during migration. Control border cells start their movement, migrate, and reach the anterior side of the oocyte (o; arrowheads). Border cells expressing Cdc42^DN^ fail to migrate. Border cell clusters (arrows) are visualized by *slbo*-GAL4-driven expression of mcD8:GFP (**B**). Quantification of the frequency of various migration outcomes of control border cells (WT, black bars) or border cells overexpressing wild-type (WT) Cdc42 (red bars) or DN Cdc42 (blue bars) in stage 10 egg chambers. Error bars represent standard deviation; n ≥ 50 egg chambers in each of 3 trials (**C**). Quantification of protrusion number (left) and lifetime (right panel) in control (n = 8 movies) and Cdc42^DN^ (n = 10 movies) border cell clusters shown as box-and-whisker plots (**D**). All data points are shown; whiskers represent the minimum and maximum measurements, the box extends from the 25th to 75th percentiles, and the line indicates the median. Statistics calculated based on an unpaired two-tailed t test (**C**) or one-way ANOVA (**D**), *p<0.05, **p<0.01, ***p<0.001.

**Supplemental Figure 4. Requirement for Cdc42 in GBM CSC migration.**  Representative micrographs of CSC-enriched spheres from a patient-derived xenograft (L1) model treated with a Cdc42 inhibitor (ML141, 200 μM) demonstrates limited migration as compared with a DMSO control (**A**). Quantification of cell viability (**B**), survival (**C**), and proliferation (**D**) of CSC-enriched spheres treated with a Cdc42 inhibitor (ML141, 200 μM) demonstrates no major changes with inhibitor treatment versus control. Values represent means +/- standard deviation (n = 4).
